# Noninvasive Characterization of Preservation Fluids through Glass Container Using Spatially Offset Raman Spectroscopy: Potential in Heritage Science

**DOI:** 10.1021/acsomega.4c11521

**Published:** 2025-02-20

**Authors:** Sara Mosca, Wren Montgomery, Chelsea McKibbin, Robert Stokes, Claudia Conti, Pavel Matousek

**Affiliations:** † Central Laser Facility, Research Complex at Harwell, STFC Rutherford Appleton Laboratory, UKRI, Harwell Campus, Didcot OX11 0QX, U.K.; ‡ Science Innovation Platforms, Department of Science, Natural History Museum, Cromwell Road, London SW7 5BD, U.K.; § Agilent Technologies LDA U.K., Becquerel Avenue, Didcot OX11 0RA, U.K.; ∥ Institute of Heritage Science, National Research Council (CNR-ISPC), Via Cozzi 53, 20125 Milan, Italy

## Abstract

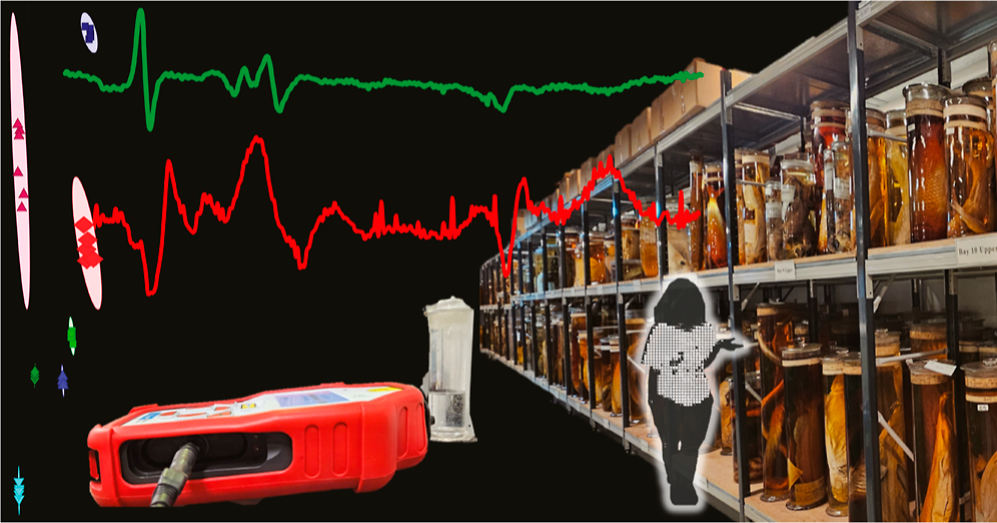

The conservation and characterization of preservation fluids are crucial for maintaining specimen integrity in natural history fluid collections. However, characterizing these fluids analytically poses significant challenges, especially as noninvasive methods are preferred to avoid opening jars and reduce the risk of compromising specimens. This proof-of-concept study investigates the feasibility of using a hand-held spatially offset Raman spectroscopy (SORS) instrument to determine the chemical composition of preservation fluids through their original glass containers. Results demonstrate that SORS can noninvasively verify the chemical identity of dominant excipients in these fluids measured through a historic glass jar. Additionally, multivariate analysis combined with SORS measurements successfully differentiated several types of typical preservation fluids prepared as mixtures of different alcohols in water, such as glycerol, ethanol, methanol, and formaldehyde. The proposed noninvasive approach was also able to differentiate between different concentration points of components in water within the same type of preservation fluid.

## 1 Introduction

The preservation of biological specimens in fluid solutions is of critical importance in heritage science, particularly for natural history collections where animals and plants, to name a few, are preserved in chemicals and research archives. These preservation fluids are often mixtures of alcohols [i.e., different concentrations of ethanol (EtOH) and methanol (MetOH)] and other chemicals such as formaldehyde and glycerol.^1^ Jars of historic fluid collections may contain a wide variety of chemicals in solid or liquid phase, some potentially toxic.^2^ These can originate from the initial fixation and preservation fluids, subsequent chemical degradation of these due to environmental conditions (i.e., temperature, humidity, and light), outside contamination, and evaporation due to the jar sealant not being optimal or the interactions between the specimen, the fixatives,^3^ and the fluid.^4,5^ Adding or transferring to different fluids or changing concentrations can harm specimen preservation,^6,7^ and rehydration of dried specimens can also present challenges.^8^ Understanding and maintaining the correct chemical composition of these fluids is essential for their optimum management and conservation. Additionally, with historical artifacts, the chemical composition of preservation fluids inside sealed containers can be unknown. Several analytical techniques are commonly used to assess the chemical composition of the preservation fluids. For example, gas chromatography coupled with mass spectrometry (GC–MS) has been successfully used to identify the preservation fluids and dissolved lipids and helped identify peptides or proteins released from specimens.^9,10^ Another method consists in measuring the density of preservative fluids (e.g., using digital density meters), enabling the identification of EtOH or formaldehyde concentrations.^11,12^ While these methods provide precise information about the composition and degradation of fluids, they require the opening of the historic jar to access the preservation liquid. This may potentially compromise the optimum conservation of the historic artifact (i.e., fluid loss, contamination, exposes the specimen to oxygen, and pollutants) and also presents personnel safety issues (e.g., risk of inhaling toxic fumes by the handler).^2^

Raman spectroscopy has emerged as a promising tool for noninvasive chemical analysis in several application fields.^13^ It is a vibrational spectroscopy technique that conveys highly molecular specific information on agents present in samples through detected inelastically scattered light, without the need for sample preparation.^14^ A recent study^12^ has demonstrated the potential of laboratory-based conventional Raman microscopy for the identification of different historic fluid recipes, providing a valuable tool for conservation science without compromising specimen integrity. This approach is still limited to laboratory-based instruments and as such applicable only to specimens that can be transported and fit inside a microscope compartment. Here, we investigate the potential of a recently developed technique, spatially offset Raman spectroscopy (SORS),^15,16^ a variant of Raman spectroscopy that enables analysis through both transparent (i.e., glass)^17^ and opaque (i.e., plastic) containers with enhanced sensitivity^18^ and can easily be implemented for in situ analysis. SORS uses a spatial separation between the collection and illumination zones on the sample surface to suppress interfering signals from the container walls and retrieve the subsurface chemical composition of the content.^16^ Since its development, it has been widely used, for example, in pharmaceutical manufacture for raw material identification through packaging^19^ and in airport security for detection of explosives.^20^ More recently, SORS was used to rapidly detect falsified COVID-19 vaccines through unopened vials^21^ and to noninvasively authenticate UK honeys.^22^ Its microscale modality, micro-SORS, has been successfully applied in Cultural Heritage studies to noninvasively reconstruct painted layer sequences,^23^ hidden texts,^24^ and figures; advanced portable micro-SORS devices have been developed for unlocking in situ measurements in museum collections and galleries.^25^ A recent study^26^ has explored the suitability of SORS and micro-SORS for characterizing stratified samples, highlighting their mutual strengths, limitations, and specific instrumental effects on cultural heritage mock-up samples. In contrast, the potential of portable SORS in cultural heritage research has not been explored to the same extent.

In this study, we employed a hand-held SORS device in combination with principal component analysis (PCA) to distinguish between different preservation fluids through sealed glass jars. This approach offers several advantages: it eliminates the need to access the fluid by opening the jar and allows for in situ analysis without transporting the sample, both of which are critically important for the intended application. The SORS method was chosen as it is less affected by interference from the container wall compared to conventional Raman spectroscopy,^18^ offering potentially higher sensitivity and higher differentiating power.^21^ The study yielded good differentiation between different concentration levels of preservation fluids as well as enabling the discrimination of different types of fluids.

## 2 Experimental Section

### 2.1 Liquid Solutions

**Figure 1:**
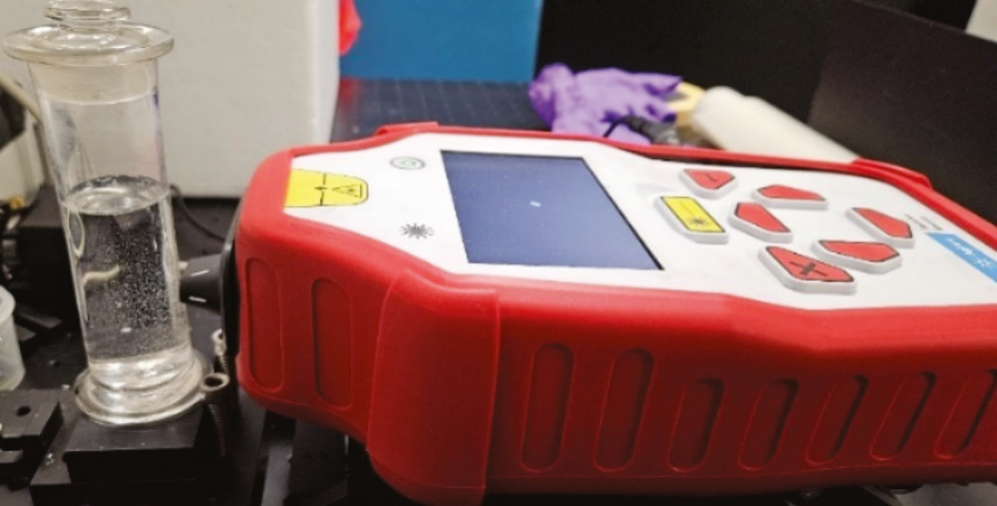
Photo of the hand-held SORS instrument and historic glass jar containing preservation fluid.

Solutions at different concentrations in ultrapure water (Milli-Q, Merck), were prepared using the following chemicals: 4% formaldehyde (EM grade, EMS), MetOH (i.e., ≥99.8%, Sigma-Aldrich), EtOH (i.e., ≥99.5%, Sigma-Aldrich), and glycerol (≥99.0%, Sigma-Aldrich). A detailed list of the concentrations tested is given in Table 1. The solutions (volume = 20 mL) were measured first through borosilicate vials (Fisherbrand) and then, sequentially, in a historic glass jar (external diameter 30 mm, height 125 mm, and glass wall thickness 3 mm; see Figure 1). The solutions were measured through the same historic jar, provided from the Natural History Museum London (pre-World War II). After each measurement, the liquid was decanted, and the jar was rinsed with deionized water, wiped dry with absorbing tissue, and allowed to air-dry in the fume hood for 30 min before proceeding with the next sample preparation. The selected concentrations represent typical concentrations that can be found in wet collections (i.e., solutions A–D and H–I) and/or simulate potential cross-contamination over time due to topping up with incorrect solutions (i.e., solutions F, G, and J).

**Table 1:** List of Solutions with Relative Concentrations Measured in This Study^a^

label	mock up solutions	main concentration
A	glycerol	5%
B	glycerol	35%
C	glycerol	65%
D	industrial methylated spirits (IMS)	EtOH 95% and MetOH 3%
E	EtOH	70%
F	EtOH and MetOH mix	EtOH 70% and MetOH 5%
G	EtOH and MetOH mix	EtOH 70% and MetOH 10%
H	formaldehyde	4%
I	formaldehyde	1%
J	formaldehyde and EtOH mix	formaldehyde 1% and EtOH 70%

a (All the solutions are water-based unless otherwise specified).

### 2.2 Instrument

Measurements were conducted using a commercial hand-held SORS device (Resolve, Agilent Technologies, Oxfordshire, UK) with an 830 nm excitation wavelength and a maximum power output of 475 mW. The instrument was operated in “through-barrier” mode by selecting the “thick, colored, or opaque” container option in the menu. SORS spectra for each sample were collected with a total acquisition time of 25 s, comprising zero spatial offset (1 s × 5 acquisitions) and a spatial offset of 5.5 mm (2 s × 10 acquisitions). The overall measurement time, including automated calibration and background checks, was approximately 1.5 min. The measurements were performed in a dark laboratory. Six measurements were conducted at different vial and jar positions for each mock-up solution. The glass containers were measured from the side (as shown in Figure 1).

### 2.3 Data Analysis

Raw SORS spectra (“zero” and “offset”) were exported from the device for external analysis. A semiautomated routine (code developed in Matlab) was used to scale subtract the zero measurement from the spatially offset one, in order to isolate a pure Raman spectrum of the jar contents. The scaling factor for the subtraction was chosen to reduce the jar fluorescence near its peak (∼675 cm^–1^) to approximately zero in the subtracted spectrum. The SORS spectra of the fluids were truncated below 750 cm^–1^ and above 1800 cm^–1^ before further multivariate analysis. PCA was conducted on the truncated spectra using Solo (Solo 8.7, Eigenvector Research Inc.) after a preprocessing routine consisting of a third order polynomial baseline removal and standard normal variable normalization (SNV). For further comparison, PCA was also performed on the SORS spectra preanalyzed internally by the instrument (this internal processing included polynomial baseline subtraction, scale subtraction of the offset, and zero measurement to remove the glass contribution from the fluid spectra). Additionally, PCA was performed on the OFFSET spectra only, i.e., raw offset data without baseline correction, comparable to a “displaced Raman”^17^ approach and the ZERO spectra only (i.e., raw zero data without baseline correction, representing container-wall measurements). These comparisons were made to evaluate the impact of preprocessing and scale subtraction routines on the PCA results, aiming to understand the practical limitations of each approach.

## 3 Results and Discussion

**Figure 2:**
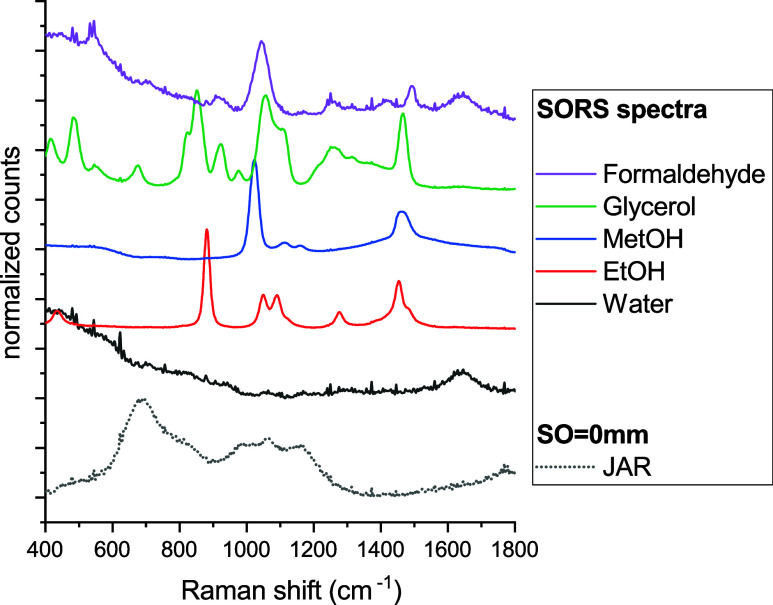
Reference Raman spectra of individual chemical components used in the study (jar and water), EtOH (70% in water), MetOH (90% in water), glycerol (65% in water), and formaldehyde (4% in water). All were measured using SORS through a glass vial. In case of the empty jar (black dotted line), a representative conventional Raman spectrum of the jar was acquired with a 0 mm spatial offset to illustrate the fluorescence interference contribution from the container wall that would be otherwise imprinted onto a conventional Raman spectrum measured through glass (as stated earlier, this component is suppressed and removed in SORS measurements).

Figure 2 presents the reference spectra of the individual components used in this study. The SORS approach effectively eliminated the Raman and fluorescence contributions from the glass jar (indicated by the gray dotted line in Figure 2) that would be present in conventional Raman measurements performed through the jar. A characteristic Raman spectrum for each alcohol within the fingerprint spectral region (i.e., 400 to 1800 cm^–1^) exhibits distinctly different spectral features, allowing clear differentiation based on their Raman profiles. Additionally, the visibility of the Raman band of water (broad band around 1640 cm^–1^, assigned to the intramolecular bending mode of the water molecule H–O–H,^27,28^ solid black line in Figure 2) is crucial, as it enables multivariate analysis to also capture the relative concentrations of the observable chemicals within the SORS spectrum of the preservation fluid.

**Figure 3:**
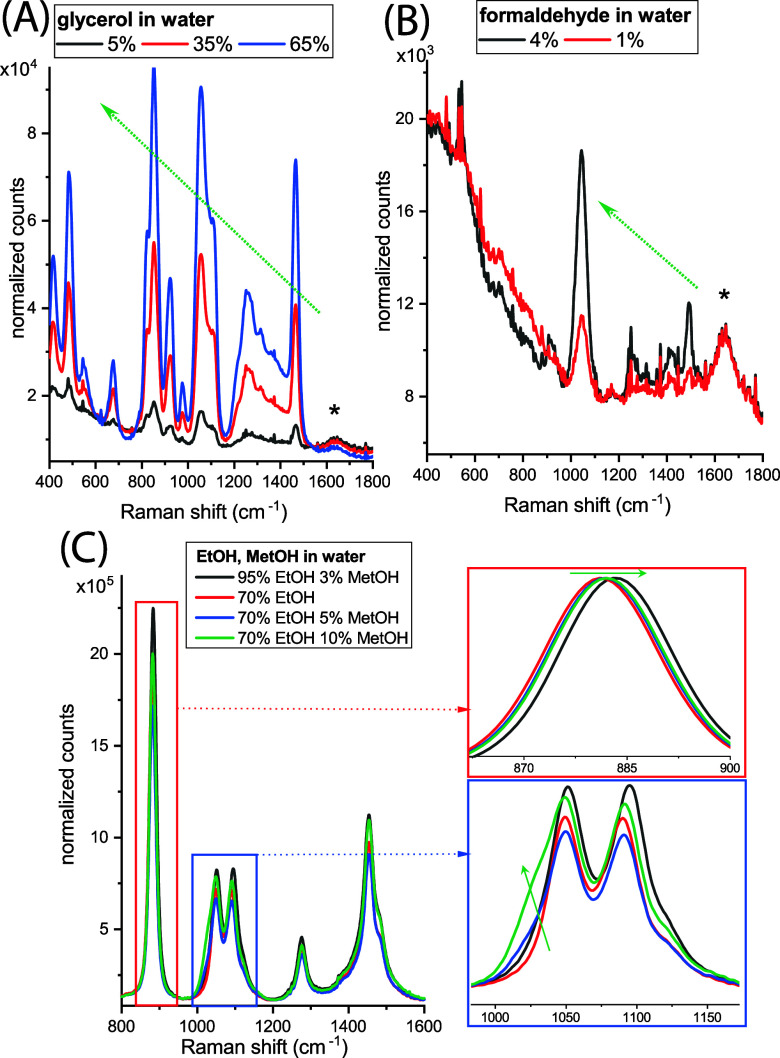
Representative SORS spectra of the mock-up solutions at different concentrations: (A) glycerol in water, (B) formaldehyde in water, and (C) EtOH and MetOH mixture in water. Black asterisk and green arrow highlight the main spectral changes that occur at different concentrations.

Representative SORS spectra of the mock-up solutions at different concentrations (Figure 3 and Table 1) highlight underlying spectral trends observed across various concentrations for each type of solution. For example, an increase in the relative intensity of the glycerol peaks (e.g., 483, 852, 1056, and 1466 cm^–1^) compared to the water component (1640 cm^–1^) can be observed with increasing glycerol concentration (Figure 3A). Similarly, relative intensity increases in the formaldehyde Raman bands (1044, 1250, and 1492 cm^–1^) in comparison to the water Raman band (1640 cm^–1^) are seen when its concentration increases from 1 to 4% (see Figure 3B). Another observation is a blue shift of the dominant Raman band of EtOH (see Figure 3C, i.e., from 883 to 879 cm^–1^) as the water concentration increases (and therefore the relative EtOH concentration decreases). This effect is due to strong hydrogen bonding between EtOH and water molecules, which weakens nearby C–C, C–O, and C–H bonds, leading to changes in molecule polarizability.^29^ Additionally, a band shoulder around 1040 cm^–1^ appears as the MetOH concentration increases in the mockup solution, as expected.

**Figure 4:**
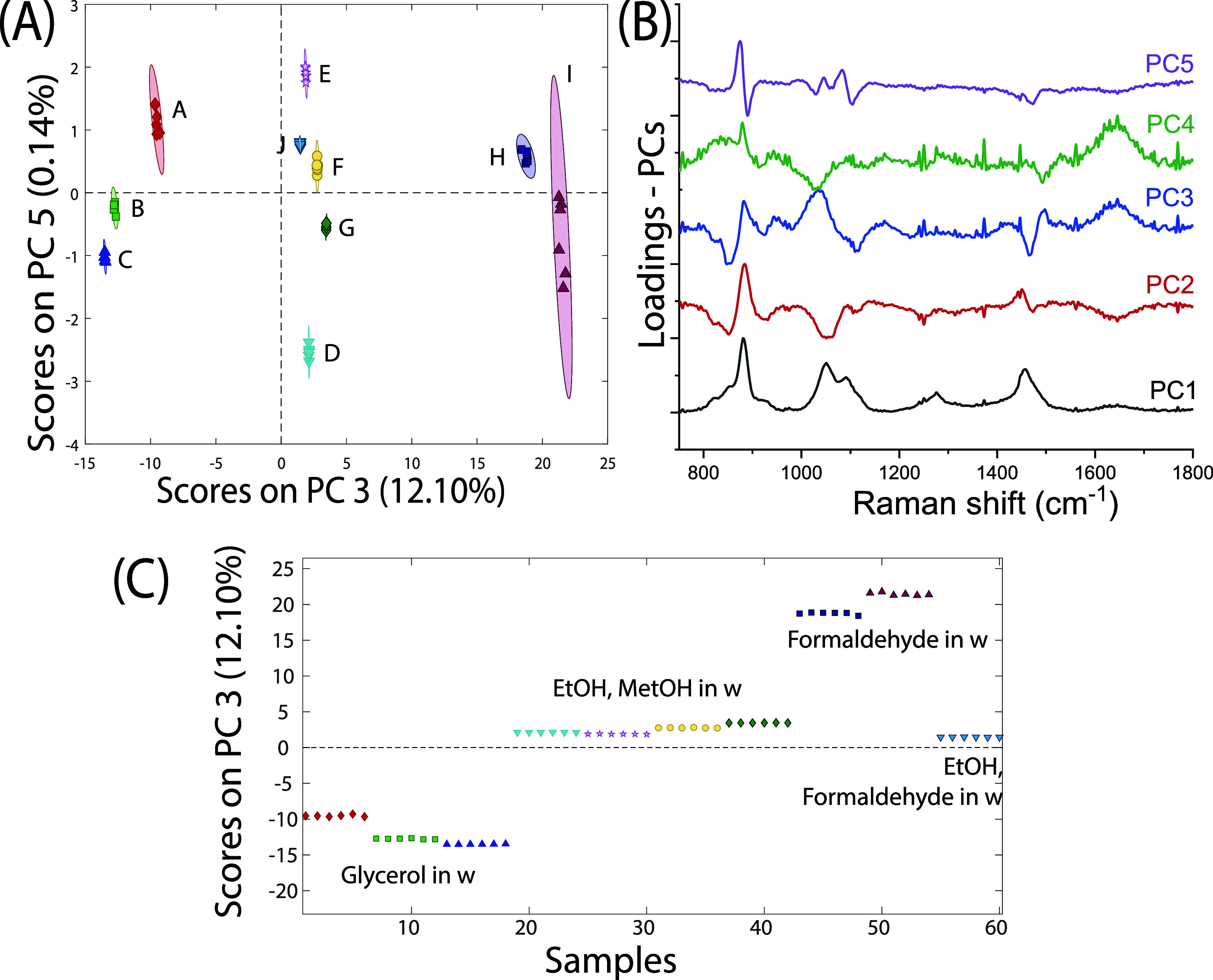
(A) PCA score plot of significant principal components showing an ability to discriminate different preservation fluids from each other (95% confidence intervals are shown). Letter labels refer to solution coding shown in Table 1. (B) Most significant PCA eigenvectors, showing how the different Raman components contribute to a particular principal component. (C) PC3 scores coefficient for the different samples highlighting three subclasses of fluids: different concentrations of glycerol in water solutions (labeled A, B, and C); EtOH, MetOH, and formaldehyde mixtures (labeled D, E, F, G, and J); and different concentrations of formaldehyde in water (labeled H and I).

PCA was performed as described above (see the Data Analysis section) on all mock-up preservation fluid solution data using all the available repetitions (Table 1). This yielded clear separation between different solutions, as shown in Figure 4A, where the PCA biplots of significant principal components are shown along with their corresponding eigenvectors (Figure 4B). The most effective separation was achieved using PC3 and PC5.

Additionally, the inspection of the scores on PC3 component (Figure 4C) shows it is possible to identify three distinct subsets of fluids: the glycerol and water mixtures (labeled A, B, and C) characterized by negative coefficients for PC3 (blue line, Figure 4B); the EtOH, MetOH, and formaldehyde mixtures in water (labeled D, E, F, G, and J) with a low value coefficient for PC3, and formaldehyde alone in water (labeled H and I) characterized by higher scores for PC3 (Figure 4C).

**Figure 5:**
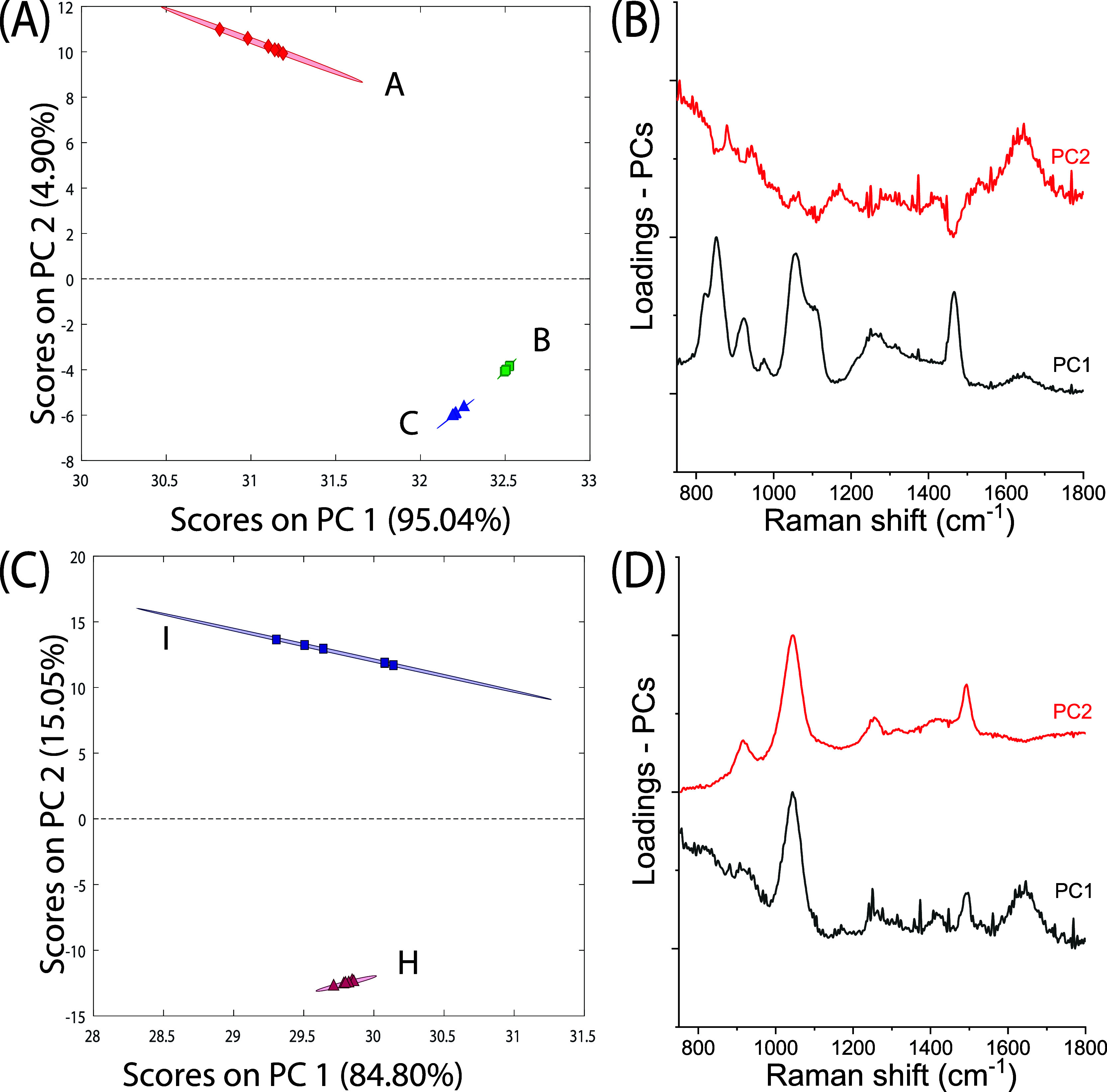
PCA results for subdata sets containing different concentrations of (A,B) glycerol in water and (C,D) formaldehyde in water. (A,C) PCA score plots of the two most significant principal components showing the ability to discriminate the different concentration solutions for each class of fluids. Letter labels refer to solution coding shown in Table 1. (B,D) Corresponding eigenvectors.

**Figure 6:**
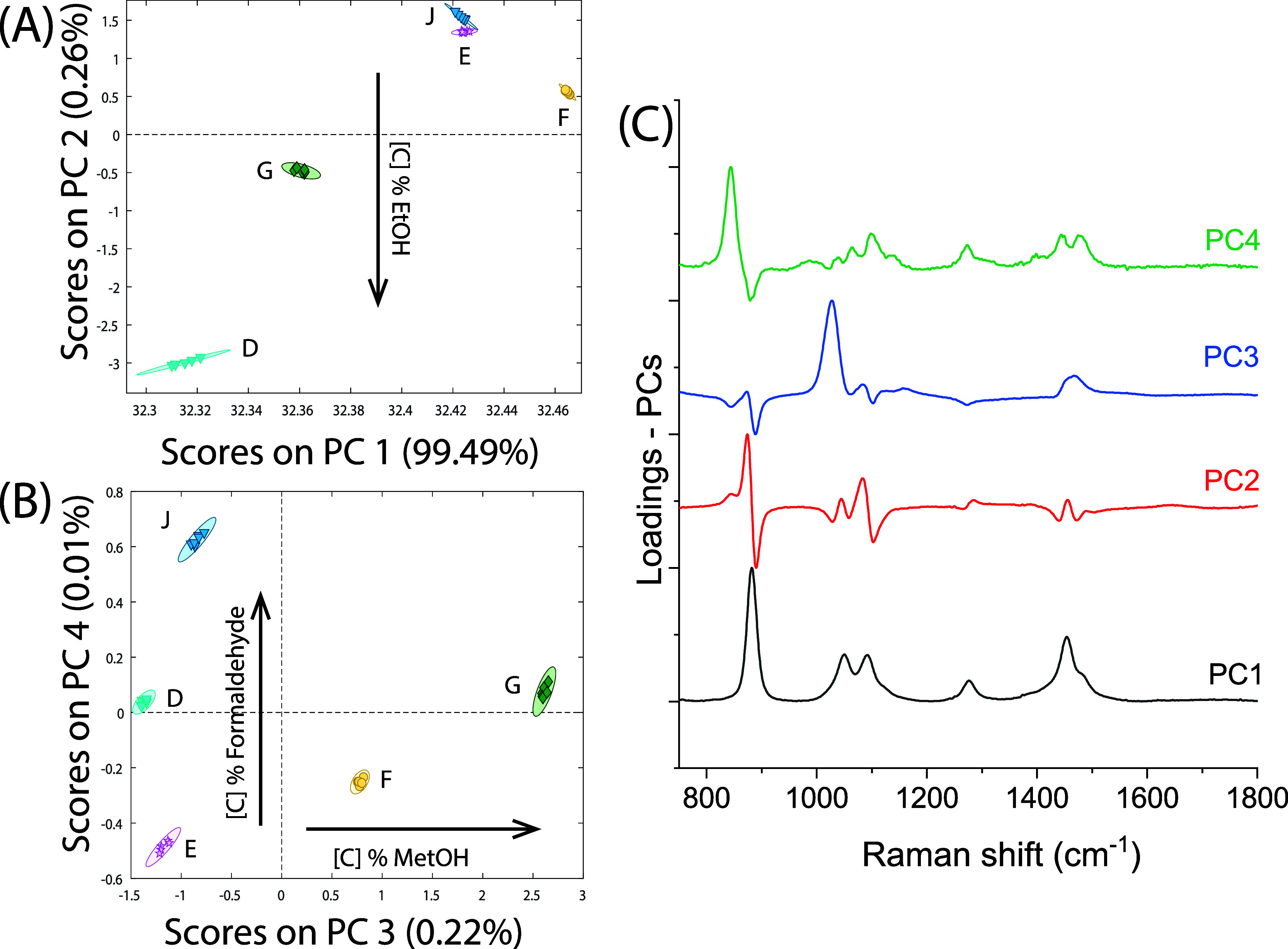
PCA results of subdata sets containing different concentration levels of EtOH mixed with MetOH and formaldehyde in water. (A,B) PCA score plots of the two most significant principal components (A) PC1 versus PC2 and (B) PC3 versus PC4 showing the ability to discriminate (A) the different concentrations of EtOH and (B) the presence of MetOH and formaldehyde at different concentrations. Letter labels refer to solution coding shown in Table 1. (C) Relevant eigenvectors evidencing that the discrimination between samples at different concentrations is based on chemical information contained in spectra.

By analyzing these three subclasses of samples separately, it is also possible to distinguish more clearly between different concentration levels of the same type of fluid. Figure 5 shows the PCA of different types of fluids. The 5, 35, and 65% glycerol in water mixtures are separated (Figure 5A) based on the water content in the mixture (red line, PC2 loading in Figure 5B). Similarly, 1 and 4% formaldehyde solutions in water are also clearly differentiated (Figure 5C) through the variation of water and formaldehyde relative band intensities (PC1 and PC2 in Figure 5D). PCA of EtOH and MetOH in water mixtures (Figure 6) reveals that SORS can effectively distinguish different EtOH concentrations (i.e., industrial methylated spirits vs 70% EtOH, labeled as D and E, respectively, in Figure 6A) through a shift in the main EtOH Raman band (red line, PC2 loading in Figure 6B). Also solutions “F” and “G” separate with respect to “E” along PC2 due to the presence of MetOH. Additionally, the increase in concentration of MetOH in the mixtures is reflected in the scores of PC3 (blue line, PC3 loading in Figure 6C), and the variation of formaldehyde content is captured in PC4 (green line, PC3 loading in Figure 6C). Figure 6B shows the PCA score plots for the PC3 versus PC4 components, where it is possible to discriminate: from left to right (corresponding to from lower to higher PC3 scores), a different concentrations of MetOH in the solution and, from bottom to top, a different concentrations of formaldehyde.

Conventional Raman spectroscopy could potentially also be used to identify preservation fluids through a glass jar; however, these measurements are typically more significantly affected by interfering fluorescence from the jar itself,^12^ which can vary depending on the container material, fluorescence emission, and thickness. In contrast, the SORS method is less influenced by these effects, making it generally a more robust solution.^18,21^

To see if the type of glass plays any significant role in fluid separation with SORS measurements, we have also performed the analysis on historic jar and modern glass vial data sets, the latter obtained by performing SORS measurements through modern glass vials. The SORS spectra obtained through different glass containers (i.e., modern glass vial and historic glass jar) were analyzed with the procedure discussed. Once again, good separation was achieved for all types of fluids (see Supporting Information, S1), demonstrating that SORS performance remains consistent, regardless of variations in the glass container used to preserve the species.

The same PCA was also carried out on the previously described data set, using different types of data extracted from the instrument to assess the impact of preprocessing and scale subtraction routines on fluid differentiation. Detailed results are provided in the Supporting Information. Specifically, the analysis of the SORS data sets preanalyzed internally by the instrument (Figure S2) demonstrates somewhat poorer performance. The method was able to discriminate between major fluid subclasses, such as glycerol, formaldehyde, and EtOH–MetOH mixtures, but this approach failed to effectively differentiate varying concentrations of the primary excipient within the same fluid (e.g., 5, 35, and 65% glycerol; 1 and 4% formaldehyde) or to detect cross-contamination (e.g., 1% formaldehyde in 70% EtOH). In contrast, the analysis of spatially OFFSET spectra only (Figure S3) yielded good results comparable to those obtained from the externally processed SORS spectra shown in Figure S1, highlighting the ability to distinguish fully different preservation fluids based on chemical composition. This effectiveness is attributed to the use of a spatial offset (i.e., “displaced Raman”) between the excitation and collection areas, which, in this case, was sufficient to suppress the fluorescence contribution from the 3 mm-thick container wall. In contrast and as expected, the analysis of ZERO spectra only (Figure S4) showed no clear differentiation between the fluids. This is because the ZERO spectra are dominated by fluorescence signals from the container (e.g., glass fluorescence) rather than the chemical information on the fluid inside the container. These findings underscore the advantages of SORS and offset (i.e., displaced) Raman approaches for addressing this specific challenge.

## 4 Conclusions

In this study, we have demonstrated the potential of a portable SORS device to noninvasively identify common preservation fluids through sealed glass jars and vials. The results indicate that SORS can accurately identify the chemical composition of primary fluid ingredients (i.e., EtOH, MetOH, glycerol, and formaldehyde) and, when combined with multivariate analysis, differentiate different concentration levels of the same type of fluid. SORS minimizes container interference (e.g., fluorescence), making it well-suited for deployment across various container types. Here, we have also demonstrated the potential of portable SORS to the cultural heritage field. This proof-of-concept study paves the way for in situ analysis across a diverse range of specimen collections.

## Data Availability

Data openly available in STFC public repository eDATA^30^ (https://edata.stfc.ac.uk/handle/edata/967).

## Supporting Information Available

Discrimination of fluids through different glass-type containers and multivariate analysis (PCA) on SORS spectra internally preanalyzed with RESOLVE, OFFSET, and ZERO spectra only (PDF)
