# Supplementary material for: Noninvasive Characterization of Preservation Fluids through Glass Container Using Spatially Offset Raman Spectroscopy: Potential in Heritage Science
Source: ACS Omega. 2025 Feb 20;10(8):8658–64. doi: 10.1021/acsomega.4c11521 (PMC11886732; doi:10.1021/acsomega.4c11521)
Supplement: Supplementary file 1 — ao4c11521_si_001.pdf [file ao4c11521_si_001.pdf]

Supplementary Information

Non-invasive characterization of preservation fluids through glass container using Spatially Offset Raman Spectroscopy: potential in heritage science

Sara Mosca[a*], Wren Montgomery[b], Chelsea McKibbin[b], Robert Stokes[c], Claudia Conti[d] and Pavel Matousek [a,d]

a Central Laser Facility, Research Complex at Harwell, STFC Rutherford Appleton Laboratory, UKRI, Harwell Campus, OX11 0QX, UK

b Science Innovation Platforms, Department of Science, Natural History Museum, Cromwell Road, London SW7 5BD, UK

c Agilent Technologies LDA UK, Becquerel Avenue, Didcot OX11 0RA, UK

d Institute of Heritage Science, National Research Council (CNR-ISPC), Via Cozzi 53, 20125, Milan, Italy

*Corresponding author: Sara Mosca sara.mosca@stfc.ac.uk

Table of contents:

S1: Discrimination through different types of glass.

S2: Multivariate analysis (PCA) on SORS spectra internally pre-analysed with RESOLVE.

S3: Multivariate analysis (PCA) on OFFSET only spectra.

S4: Multivariate analysis (PCA) on ZERO only spectra.

S1: **Discrimination of different preservation fluids measured through different glass containers.**

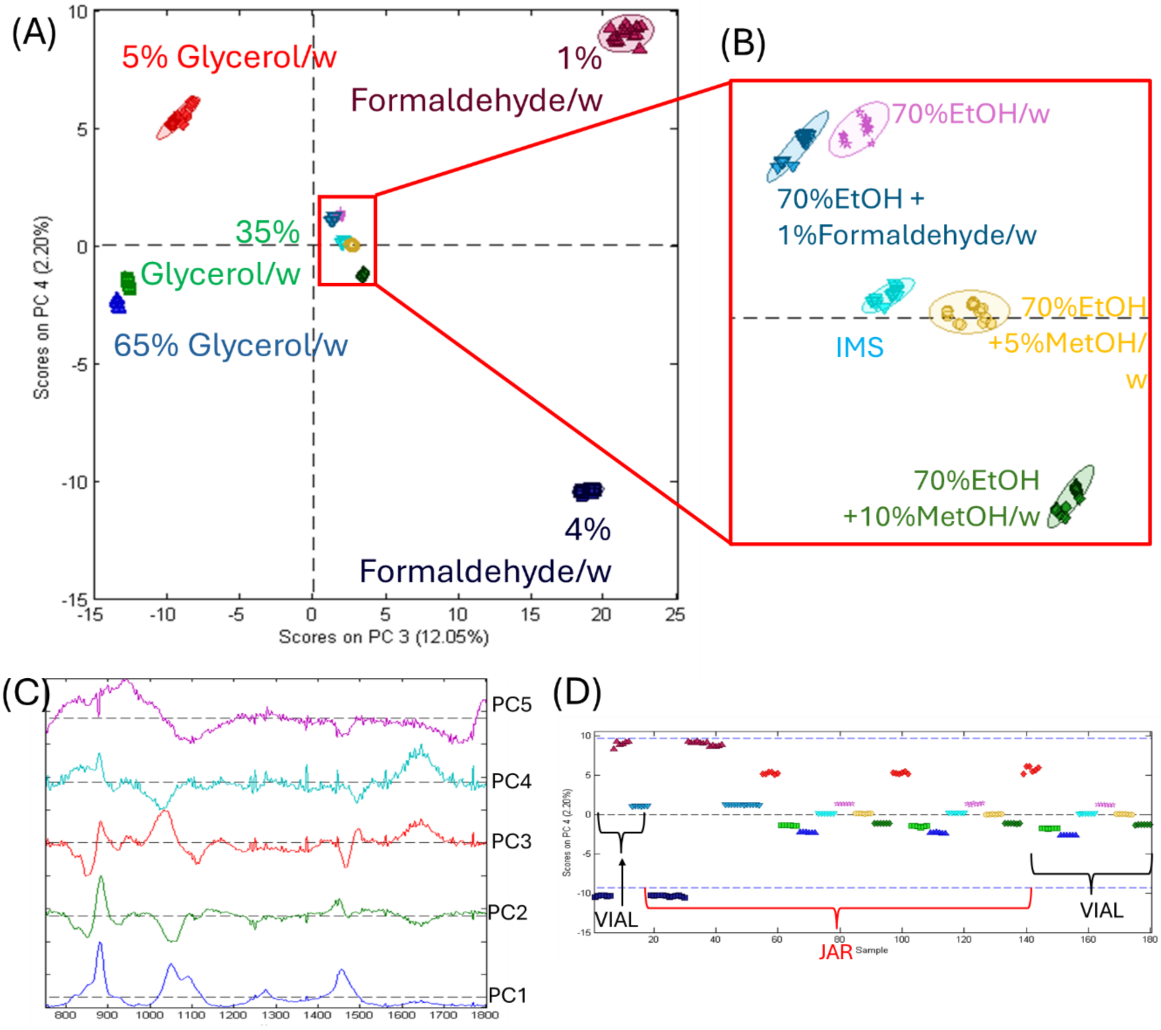

Figure S1: (A) PCA score plot of significant principal components showing ability to discriminate different preserving fluids from each other (95% confidence intervals are shown) by analysing them through both types of glass (i.e. vials and historic jar). Different colours represent different fluids. Note both types of glass are shown in the same colour (plotted within the same 95% confidence interval). (B) Zoom insert of the PCA score plot. (C) The most significant PCA eigenvectors, highlighting how the different Raman components contribute to a particular principal component. (D) PC4 score coefficients for the different samples highlighting good differentiation based on the chemical information present in the loading. Labels 'VIAL' and 'JAR' highlight the container used for the different SORS repetitions. (measurement were performed on different days).

S2: **Multivariate analysis (PCA) on SORS spectra internally pre-analysed with RESOLVE**

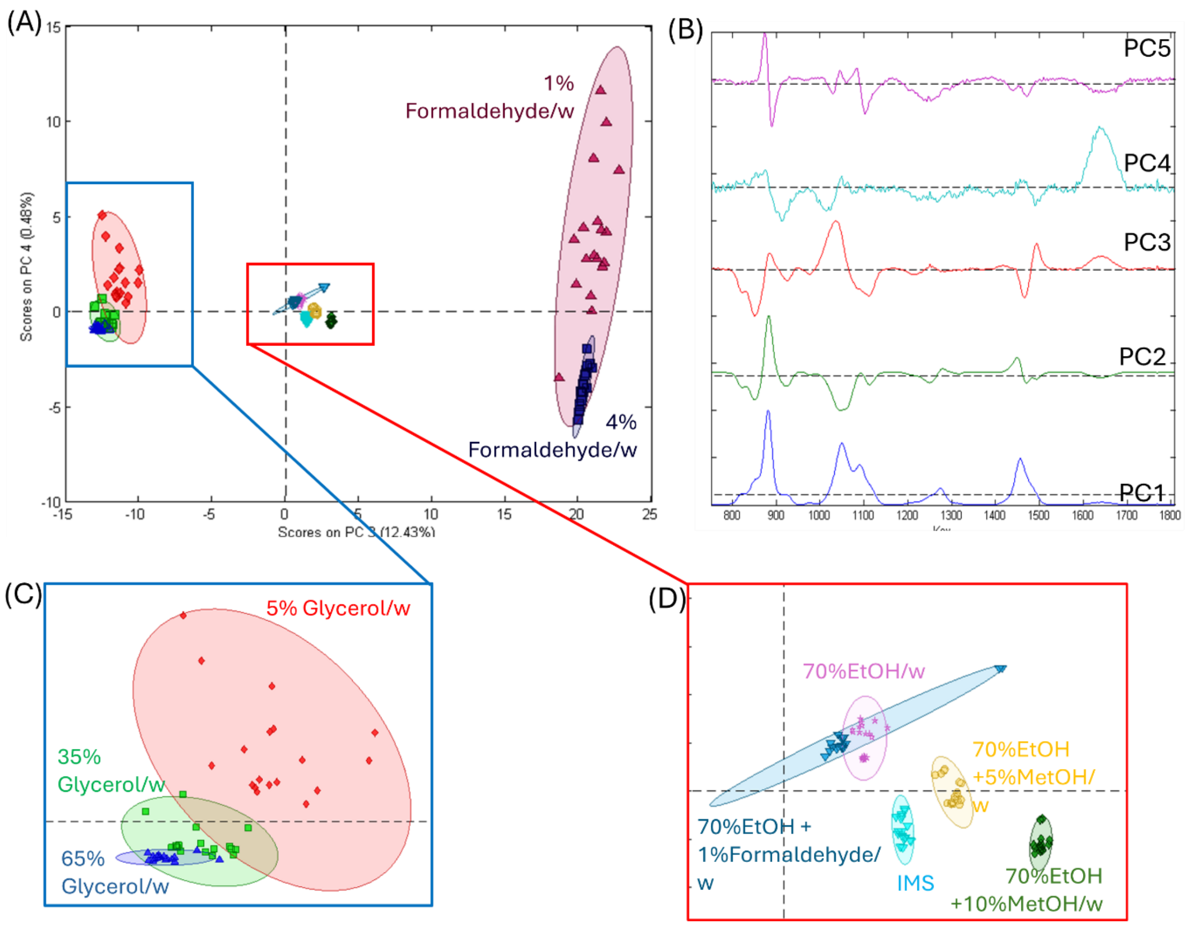

Figure S2: Multivariate analysis performed on the same dataset described above (see S1), consisting of all fluids measurements through both types of glass (i.e. vials and historic jar), where the SORS spectra were pre-processed by Resolve instrument (i.e. baseline removal, scale subtraction of the offset and zero measurements for removal of the container contribution). (A) PCA score plot of significant principal components (i.e. PC3 vs PC4). Different colours represent different fluids/fluid mixtures (95% confidence intervals are shown). (B) The most significant PCA eigenvectors. (C-D) Zoom insert of the PCA score plot. (C) A zoom on different concentrations of glycerol in water (D) EthOH, MetOH and formaldehyde mixture.

Analysis on this SORS datasets (pre-processed and scale subtracted by Resolve instrument) show the capability of discriminating the main subclasses of fluids such as the one containing: 1. glycerol, 2. Formaldehyde 3. Ethanol 4. Ethanol and methanol mixtures from each other. However, the method did not effectively differentiate between varying concentrations of the primary excipient within the same fluid (e.g., 5%, 35%, and 65% glycerol; 1% and 4% formaldehyde) nor it detected the presence of cross-contamination (e.g., 1% formaldehyde in 70% ethanol).

S3: **Multivariate analysis (PCA) on OFFSET only spectra – "displaced Raman"**

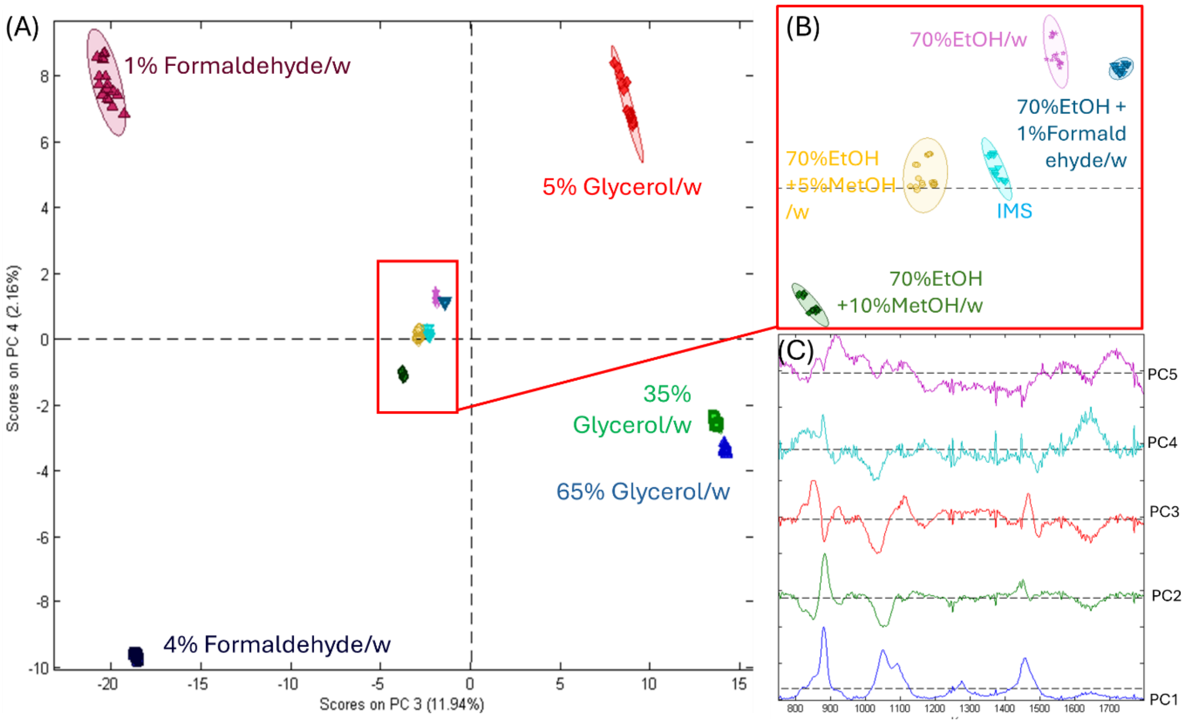

Figure S3: Multivariate analysis performed on dataset consisting of all fluids measurements through both types of glass (i.e. vials and historic jar), where only the spatially offset spectra were extracted from the Resolve instrument and used in the analysis directly (i.e. unbaselined with the zero spatial offset spectrum not being used for container signal subtraction) (A) PCA score plot of significant principal components (PC3, PC4) showing ability to discriminate different preserving fluids from each other (95% confidence intervals are shown) by analysing them through different types of glass (i.e. vial and historic jar). Different colours represent different fluids. Note both types of glass are shown in the same colour (plotted within the same 95% confidence interval). (B) Zoom insert of the PCA score plot. (C) The most significant PCA eigenvectors, highlighting how the different Raman components contribute to a particular principal component.

Analysis on the spatial offset only data (see Fig. S4) yielded comparable results to those obtained from the externally process SORS spectra (see Fig. S1), demonstrating the ability to distinguish between different preserving fluids based on their chemical composition. This effectiveness is attributed to the use of a spatial offset (i.e. displacement) between the excitation and the collection area, which alone was good enough to suppress the fluorescence component originating from the container.

S4: **Multivariate analysis (PCA) on ZERO only spectra –** surface information, container interference**.**

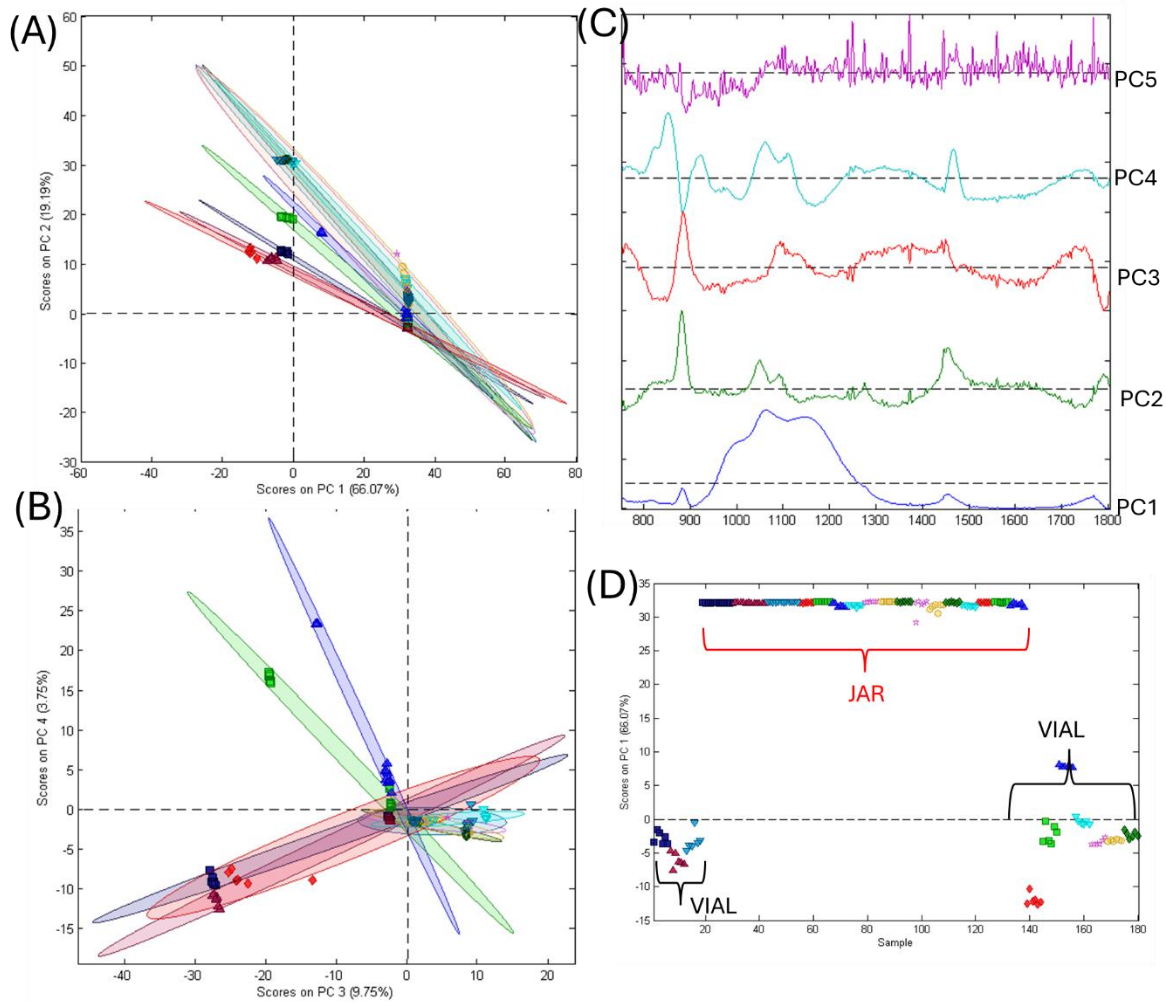

Figure S4: Multivariate analysis performed on dataset consisting of all fluids measurements through both types of glass (i.e. vials and historic jar), where only the ZERO measurements were extracted from the Resolve instrument (i.e. unbaselined) and used directly in the analysis (A-B) PCA score plot of significant principal components: (A) PC1 and PC2 (B) PC3 and PC4. (C) The most significant PCA eigenvectors (D) PC1 score coefficient for the different samples highlighting that the main/only evident differentiation is between the container used ('VIAL' and 'JAR') on the different fluorescence emission from the glass (see PC1 loading).

The analysis of the zero-offset dataset shows no clear differentiation between the fluids. This is as expected because the zero spectra are dominated by the fluorescence signal from the container (e.g., glass fluorescence) rather than the chemical information on the fluid contents. This is further supported by examining the PC1 loadings and scores, which indicate separation based on the type of glass container rather than the fluids themselves.
